# Performance of the Systemic Lupus Erythematosus Risk Probability Index (SLERPI) in a cohort of Colombian population

**DOI:** 10.1007/s10067-024-07108-x

**Published:** 2024-09-07

**Authors:** Mariana Celis-Andrade, Manuel Rojas, Yhojan Rodríguez, Juan Benjamín Calderon, Mónica Rodríguez-Jiménez, Diana M. Monsalve, Yeny Acosta-Ampudia, Carolina Ramírez-Santana

**Affiliations:** 1 Center for Autoimmune Diseases Research (CREA), School of Medicine and Health Sciences, Universidad del Rosario, Carrera 24 # 63-C- 69, 110010 Bogota, D.C Colombia; 2 Division of Rheumatology, Allergy and Clinical Immunology, University of California, Davis, USA; 3 Department of Internal Medicine, University Hospital, Fundación Santa Fe de Bogota, Bogota, D.C Colombia

**Keywords:** Autoimmune diseases, Lupus erythematosus, Polyautoimmunity, SLERPI, Systemic

## Abstract

**Objective:**

To evaluate the performance of the Systemic Lupus Erythematosus Risk Probability Index (SLERPI) in Colombian patients with systemic lupus erythematosus (SLE).

**Methods:**

The Colombian cohort included 435 SLE patients and 430 controls with other autoimmune diseases (ADs). Clinical and serological data were collected, and SLE was indicated by SLERPI scores > 7. The American College of Rheumatology (ACR)-1997, Systemic Lupus International Collaborating Clinics (SLICC)-2012, and European League Against Rheumatism (EULAR)/ACR-2019 criteria were used as reference standards. The impact of overt polyautoimmunity (PolyA) on SLERPI performance was assessed. Additionally, multivariate lineal regression analysis was performed to evaluate the contribution of SLERPI features to the overall SLERPI score.

**Results:**

SLE patients had higher SLERPI scores (P < 0.0001), with almost 90% meeting "definite" lupus criteria. Main factors influencing SLERPI included immunological disorder (β:44.75, P < 0.0001), malar/maculopapular rash (β:18.43, P < 0.0001), and anti-nuclear antibody positivity (β:15.65, P < 0.0001). In contrast, subacute cutaneous lupus erythematosus/discoid lupus erythematosus (β:2.40, P > 0.05) and interstitial lung disease (β:-21.58, P > 0.05) were not significant factors to the overall SLERPI score. SLERPI demonstrated high sensitivity for SLE, both for the overall SLE group and for those without overt PolyA (95.4% and 94.6%, respectively), but had relatively low specificity (92.8% and 93.7%, respectively). The model showed high sensitivity for hematological lupus (98.8%) and lupus nephritis (96.0%), but low sensitivity for neuropsychiatric lupus (93.2%). Compared to the ACR-1997, SLICC-2012 and EULAR/ACR-2019 criteria, SLERPI yielded the highest sensitivity and lowest specificity.

**Conclusion:**

SLERPI efficiently identified SLE patients in a Colombian cohort, showing high sensitivity but low specificity. The model effectively distinguishes SLE patients, even in the presence of concurrent overt PolyA.

**Table:** 

**Key Points** •*SLERPI has a high sensitivity, but low specificity compared to ACR-1997, SLICC-2012 and EULAR/ACR-2019 criteria in the Colombian population.* •*Within the SLERPI score, immunological disorder, malar/maculopapular rash, and anti-nuclear antibody positivity are the strongest predictors of SLE.* •*SLERPI model can efficiently distinguish patients with SLE, regardless of concomitant overt PolyA.* •*SLERPI demonstrates high sensitivity in identifying hematological and nephritic subphenotypes of SLE.*

**Supplementary Information:**

The online version contains supplementary material available at 10.1007/s10067-024-07108-x.

## Introduction

Historically, diagnostic classification criteria have been invaluable, not only for providing critical insights that enhance diagnostic processes but also for contributing to more accurate prognosis and informed treatment strategies [1]. Currently, a variety of clinical and laboratory criteria are used to achieve a high level of diagnostic confidence for Systemic Lupus Erythematosus (SLE). These include the American College of Rheumatology (ACR) 1997 criteria [2], the Systemic Lupus International Collaborating Clinics (SLICC) 2012 criteria [3], and the European League Against Rheumatism (EULAR) with ACR 2019 criteria [4].

Recently, Adamichou, et al. [5] developed and validated a simple and clinically applicable model, the SLE Risk Probability Index (SLERPI), for diagnosing SLE using machine learning strategies in a European population. This model incorporated 14 clinical and serological features, each assigned different weights, to classify patients as having SLE if their score exceeds 7. The model showed a sensitivity of 94.2%, a specificity of 94.4%, and an overall accuracy of 94.2%. Other researchers have validated this model in different populations. In an Australian cohort, the SLERPI demonstrated a sensitivity of 98.5%, specificity of 84.6% and accuracy of 91.5% [6]. In a Chinese population, the model showed a sensitivity of 98.3%, specificity of 89.4%, and accuracy of 93.6% [7]. Given the variations in SLERPI performance across different racial groups, further validation of this model is warranted in other populations, including Latino cohorts.

Although a Colombian study has evaluated the correlation between SLERPI scores and established classification criteria, formal validation of the model in our population has not yet been conducted [8]. Therefore, we aimed to assess the performance of SLERPI in a cohort of Colombian patients with SLE compared to a control group with other autoimmune diseases (ADs). We also assessed the influence of overt polyautoimmunity (PolyA) on SLERPI's performance in both groups.

## Methods

### Study design and participants

We conducted a case–control study involving 435 Colombian patients with SLE and a control group of 474 patients, randomly selected from a database of 2,149 individuals. To accurately evaluate the performance of the SLERPI for diagnosing SLE, we excluded controls with overt PolyA due to SLE (n: 44). Thus, the final control group consisted of 430 patients, categorized as follows: rheumatoid arthritis (RA) (n: 253), Sjögren's syndrome (SS) (n: 56), autoimmune thyroid disease (AITD) (n: 43), multiple sclerosis (MS) (n: 47), systemic sclerosis (SSc) (n: 30), and anti-phospholipid syndrome (APS) (n: 1). All subjects were followed in a cohort at the Center for Autoimmune Diseases Research (CREA) in Bogota, Colombia. This study adhered to Act 008430/1993 of the Ministry of Health of the Republic of Colombia, which classifies it as minimal-risk research. The study design was approved by the institutional Review Board of Universidad del Rosario.

### Eligibility criteria

Our study was composed of two groups. The first group, referred to as cases, included patients with a confirmed diagnosis of SLE made by expert rheumatologists with over 5 years of experience. This group also included patients with SLE who had overt PolyA (i.e., more than one AD in the same patient). The second group, or control group, consisted of patients diagnosed with other ADs that could share clinical characteristics with SLE (i.e., RA, MS, SSc, SS, APS, and AITD), but without overt PolyA given by SLE. These patients were selected through simple randomization from the main database. We applied the following exclusion criteria: (1) individuals under the age of 18, (2) pregnant or lactating individuals, and (3) individuals with declared disabilities.

### Data collection and definitions

Demographic, clinical, and laboratory data associated with the classification criteria were extracted from an secure electronic database, as described elsewhere [9]. We extracted variables related to the following classification criteria: ACR-1997 [2], SLICC-2012 [3], and EULAR/ACR-2019 [4]. Definitions for each item of the classification criteria were followed as specified for each respective scale [2–5]. Data from the 14 features of SLERPI were extracted according to the definitions provided by the authors [5], and the simplified score (> 7 points) was calculated based on the criteria outlined in the original manuscript. Additionally, other demographic and clinical data has been included, such as age, gender, age of symptoms onset and diagnosis, and the presence of overt PolyA. Based on the ordinal SLERPI score, four diagnostic probability categories were defined: (1) Definite: 87–100%; (2) Likely: 44–86%, (3) Possible: 15–43%; and (4) Unlikely: 0–14% [5]. Lupus nephritis was diagnosed based on kidney histological findings indicative of SLE in a conjunction with compatible clinical and/or serological evidence [5]. Hematological SLE was defined by the presence of any of the following: thrombocytopenia, leucopenia, or anemia. Neuropsychiatric lupus was classified according to the clinical records of each patient and assessed by a multidisciplinary treatment team [10]. The study adhered to the Declaration of Helsinki. To ensure data anonymity, all potential identifiers were removed from the database.

### Statistical analysis

Univariate descriptive statistics were performed. Categorical variables were analyzed using frequencies, while continuous variables were reported as median and interquartile range (IQR). Normality of numerical variables was assessed using Kolmogorov–Smirnov tests (i.e., n ≥ 50) and reported accordingly [11]. None of the included parameters were subjected to statistical transformation or normalization. Bivariate analyses were conducted using the Mann–Whitney U test for non-normally distributed data and the Chi-Square test for categorical variables. The Receiver Operating Characteristic (ROC) curve was constructed, and the Area Under the Curve (AUC) was estimated using a 50% cutoff point for total SLERPI scores with the pROC package. Additionally, confusion matrices were generated using the established cutoff points for each classification criterion. Sensitivity, specificity, and accuracy were estimated using the epiR package. These analyses were conducted for individuals with and without overt PolyA in SLE, and also for subgroups based on specific phenotypes, including lupus nephritis, neuropsychiatric lupus, and hematological lupus. Furthermore, multiple linear regression analysis was conducted to assess the most significant SLERPI variables in our population. The analyses were performed using GraphPad Prism V10 and R version 4.1.2.

## Results

### Overall performance of SLERPI in the classification of SLE

A total of 435 SLE patients and 430 controls were included in this study. The general characteristics of the patients are detailed in Table 1. Compared to the control group, the SLE group had a higher proportion of women (91.3%) and younger patients, with a median age of 48 years (IQR: 37.0–59.0). Additionally, the SLE group showed an earlier age of onset (median 26.5 years, IQR: 19.0–36.0) and age at diagnosis (median 28.5 years, IQR: 22.0–40.0). Moreover, this group had a frequency of 18.4% of overt PolyA, mainly associated with AITD, SS, APS and RA, as detailed in Supplementary Table 1. The control group also had an overall frequency of 18.4% of overt PolyA, being AITD (41.9%), SSc (40.0%), and SS (25.0%) the ADs index with the highest frequency of overt PolyA.

**Table 1:** Characteristics of SLE and control groups

	SLE group (n: 435)	Control Group (n: 430)	*P*-value^a^	KS P-value SLE group	KS *P*-value Control group
Demographics					
Gender (Female, %)	397 (91.3%)	366 (85.1%)	0.006	-	-
Age (Years, Median – IQR)	48.0 (37.0 – 59.0)	64 (54.0 – 70.5)	< 0.001	< 0.001	< 0.001
Age at diagnosis (Years, Median – IQR)	28.5 (22.0—40.0)	44.0 (33.5 – 53.0)	< 0.001	< 0.001	< 0.001
Age at onset (Years, Median – IQR)	26.5 (19.0—36.0)	40.0 (29.0 – 50.0)	< 0.001	< 0.001	< 0.001
Overt polyautoimmunity	80 (18.4%)	79 (18.4%)	1.000	-	-
Index Disease (n, %)			-	-	-
Systemic lupus erythematosus	435 (100.0%)	-			
Rheumatoid arthritis	-	253 (58.8%)			
Sjögren’s syndrome	-	56 (13.0%)			
Autoimmune thyroid disease	-	43 (10.0%)			
Multiple sclerosis	-	47 (10.9%)			
Systemic sclerosis	-	30 (7.0%)			
Antiphospholipid syndrome	-	1 (0.2%)			
SLERPI Feature					
Malar/maculopapular rash	209 (48.0%)	1 (0.2%)	< 0.001		
SCLE/DLE	64 (14.7%)	0 (0.0%)	< 0.001		
Alopecia	229 (52.6%)	13 (3.0%)	< 0.001		
Mucosal ulcers	170 (39.1%)	1 (0.2%)	< 0.001		
Arthritis	325 (74.7%)	290 (67.4%)	0.020		
Serositis	144 (33.1%)	0 (0.0%)	< 0.001		
Neurological disorder	88 (20.2%)	14 (3.3%)	< 0.001		
Leucopenia	175 (40.2%)	32 (7.4%)	< 0.001		
Thrombocytopenia/AIHA	45 (10.3%)	6 (1.4%)	< 0.001		
Proteinuria	124 (28.5%)	2 (0.5%)	< 0.001		
ANA	406 (93.3%)	253 (58.8%)	< 0.001		
Low C3 and C4	169 (38.9%)	7 (1.6%)	< 0.001		
Immunological disorder	379 (87.1%)	53 (12.3%)	< 0.001		
Interstitial lung disease	0 (0.0%)	2 (0.5%)	0.247		

^a^*P *values for continuous variables estimated by Mann–Whitney U test based on KS normality test. *ANA* Anti-nuclear antibodies, *C* Complement, *KS* Kolmogorov–Smirnov, *SCLE/DLE* Subacute cutaneous lupus erythematosus/discoid lupus erythematosus, *SLERPI* Systemic Lupus Erythematosus Risk Probability Index, *SLE* Systemic lupus erythematosus

**Fig. 1:**
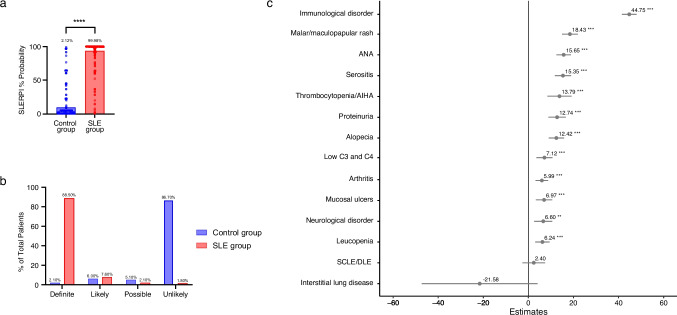
Overall performance of SLERPI in SLE and control groups.** a**. SLERPI total scores the SLE and control groups, analyzed using the Mann–Whitney U test. **b**. Distribution of SLE probability by ordinal SLERPI score. **c**. β coefficients of linear regression for SLERPI score based on clinical and serological features. Statistical significance is indicated as follows: ** *P* < 0.005, *** *P* < 0.0005, **** *P* < 0.0001, as determined by regression model. *ANA* Anti-nuclear antibodies, *AIHA* Autoimmune hemolytic anemia, *C* Complement, *SCLE/DLE* Subacute cutaneous lupus erythematosus/discoid lupus erythematosus, *SLERPI* Systemic Lupus Erythematosus Risk Probability Index, *SLE* Systemic lupus erythematosus

As expected, the SLERPI scores were significantly higher in the SLE group (median 99.98%, IQR: 98.93–100.0) compared to the control group (median 2.12%, IQR: 0.06–4.40) (Fig. 1a). According to the ordinal scale for SLERPI, patients in the SLE group were classified as definite (88.50%), likely (7.60%), possible (2.10%), and unlikely; with the latter category comprising only 1.80% of the patients (Fig. 1b). Nine patients from the control group, diagnosed with RA, AITD, or SS, were misclassified as having definite SLE by SLERPI. Among the 14 clinical and serological features evaluated, immunological disorder (β:44.75, P < 0.0001), malar/maculopapular rash (β: 18.43, P < 0.0001), and anti-nuclear antibody (ANA) positivity (β: 15.65, P < 0.0001) were highly associated with the SLERPI score (Fig. 1c). On the other hand, while the β coefficient for subacute cutaneous lupus erythematosus/discoid lupus erythematosus (SCLE/DLE) showed a tendency to be associated with the SLERPI score, it did not emerge as a significant factor in the model (β:2.40, P > 0.05) (Fig. 1c). Similarly, interstitial lung disease (ILD) as a single non-criteria feature appeared to be a potential differential factor for excluding SLE, but it did not reach statistical significance in the multivariate model (β:-21.58, P > 0.05) (Fig. 1c).

### SLERPI sensitivity and accuracy in SLE classification

**Fig. 2:**
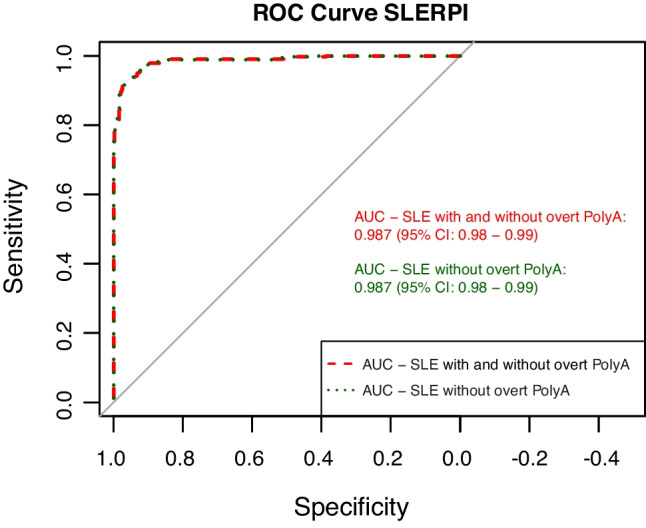
ROC curve and AUC of SLERPI score. AUC of SLERPI in SLE patients with and without overt PolyA and SLE without overt PolyA, compared to control group. *AUC* Area under the curve, *ROC* Receiver operating characteristic, *SLE* Systemic lupus erythematosus, *SLERPI* Systemic Lupus Erythematosus Risk Probability Index

To evaluate the overall performance of the SLERPI classification model, we analyzed two sub-groups: (1) SLE patients with and without overt PolyA, and (2) SLE patients without overt PolyA. This approach demonstrated that the SLERPI model efficiently distinguishes patients with SLE, regardless of concomitant overt PolyA. The AUC for both sub-groups was 0.987 (95% CI 0.98–0.99) (Fig. 2). In addition, we tested the SLERPI simplified score (> 7 points) and compared it with the nominal scores of the ACR-1997, SLICC-2012, and EULAR/ACR-2019 classification criteria (Table 2). SLERPI demonstrated higher sensitivity (95.4%, 95% CI 93.0–97.2), lower specificity (92.8%, 95% CI 89.9–95.0) and similar accuracy (94.1%, 95% CI 92.3–95.6) in SLE patients with and without overt PolyA, compared to other classification criteria (Table 2). Likewise, in the SLE patients without overt PolyA, SLERPI demonstrated higher sensitivity (94.6%, 95% CI 91.8–96.7), lower specificity (93.7%, 95% CI 90.7–96.0) and similar accuracy (94.2%, 95% CI 92.2–95.8) compared to other classification criteria. In addition, the SLERPI sensitivity slightly decreased, while specificity slightly increased in SLE patients when overt PolyA was excluded. Similar results were observed with other classification criteria, where sensitivity decrease in SLE patients without overt PolyA, while specificity remained unchanged (Table 2).

**Table 2:** Overall performance of ACR-1997, SLICC-2012, EULAR/ACR-2019 criteria, and SLERPI by overt PolyA

	Sensitivity % (95%CI)	Specificity % (95%CI)	Accuracy % (95%CI)
SLE with and without overt PolyA (n: 435)			
ACR-1997	91.0 (87.9 – 93.5)	98.8 (97.3 – 99.6)	94.9 (93.2 – 96.3)
SLICC-2012	94.5 (91.9 – 96.4)	97.2 (95.2 – 98.5)	95.8 (94.3 – 97.1)
EULAR/ACR-2019	92.4 (89.5 – 94.7)	93.3 (90.5 – 95.4)	92.8 (90.9 – 94.5)
SLERPI	95.4 (93.0 – 97.2)	92.8 (89.9 – 95.0)	94.1 (92.3 – 95.6)
SLE without overt PolyA (n: 355)			
ACR-1997	90.4 (86.9 – 93.3)	98.8 (97.3 – 99.6)	95.0 (93.3 – 96.4)
SLICC-2012	93.2 (90.1 – 95.6)	97.2 (95.2 – 98.5)	95.4 (93.7 – 96.8)
EULAR/ACR-2019	91.5 (88.2 – 94.2)	93.3 (90.5 – 95.4)	92.5 (90.4 – 94.2)
SLERPI	94.6 (91.8 – 96.7)	93.7 (90.7 – 96.0)	94.2 (92.2 – 95.8)

*ACR* American College of Rheumatology, *CI* Confidence interval, *EULAR* European League Against Rheumatism, *SLE* Systemic lupus erythematosus, *SLERPI* Systemic Lupus Erythematosus Risk Probability Index, *SLICC* Systemic Lupus International Collaborating Clinics, *PolyA* Polyautoimmunity

### SLERPI performance in SLE patients by subphenotypes

SLERPI demonstrated high performance in detecting hematological and nephritic subphenotypes (Table 3). However, in patients with neuropsychiatric lupus, sensitivity decreased and fell below the rates observed in the overall SLE group (Table 2). Specificity remained consistent across all subphenotypes evaluated.

**Table 3:** Overall performance of SLERPI by clinical subphenotypes

	Sensitivity % (95%CI)	Specificity % (95%CI)	Accuracy % (95%CI)
Neuropsychiatric lupus (n: 88)	93.2 (85.7 – 97.5)	92.8 (89.9 – 95.0)	92.9 (90.3 – 94.9)
Lupus nephritis (n: 125)	96.0 (90.9 – 98.7)	92.8 (89.9 – 95.0)	93.5 (91.1 – 95.4)
Hematological lupus (n: 173)	98.8 (95.9 – 99.9)	92.8 (89.9 – 95.0)	94.5 (92.4 – 96.2)

*CI* Confidence interval

## Discussion

This retrospective study evaluated the performance of SLERPI in a single-center Colombian cohort and compared it with the ACR-1997, SLICC-2012, and EULAR/ACR-2019 classification criteria [2–4]. SLERPI demonstrated high sensitivity for diagnosing and classifying SLE, with accuracy comparable to the other criteria. In recent years, there has been a growing effort to achieve earlier diagnosis and classification of SLE, leading to the development of new classification criteria. Due to the absence of specific diagnostic criteria, these classification criteria, originally designed to standardize disease populations for clinical study enrollment and epidemiological studies, are widely utilized as diagnostic aids [12]. The classification criteria have progressively improved, addressing its limitations. Although ANA positivity was not mandatory for classification, the ACR-1997 criteria accredited the over-representation of certain organs and domains. In the context of nephritis, the SLICC-2012 introduced the idea of "organ-dominant" disease. More lately, the EULAR/ACR-2019 criteria improved specificity by incorporating weighted manifestations, addressing the over-representation of specific organs and domains [13].

Several authors have compared the performance of the three classification criteria for SLE in different populations [13–16]. Overall, SLICC-2012 and EULAR/ACR-2019 have better diagnostic ability than the ACR-1997. Adamichou et al. [13] assessed the three existing categorization criteria in a cohort of patients with early-detected SLE. They discovered that between 25.6% and 30.5% of patients were not recognized at the time of diagnosis. The authors suggested that merging criteria sets or adjusting classification algorithms could enhance sensitivity, and this would enable the early diagnosis and treatment of individuals who are at risk of acquiring more severe disease.

Patients who did not match the ACR-1997 criteria had a notably greater prevalence of hematological and immunological characteristics. Patients who were not categorized according to the EULAR/ACR-2019 criteria showed higher frequencies of mucocutaneous disease and leucopenia, while those who were not identified by the SLICC-2012 criteria predominantly had skin and joint disease. While the use of all three classification sets allows for the early identification of a greater number of patients, a notable percentage (7–17%) with potentially serious or life-threatening illness still goes undetected or faces delays in diagnosis. Moreover, of the patients who did not meet any of the criteria, 20% displayed neurological symptoms [13].

Therefore, in 2021 Adamichou et al. [5] developed a novel model entitled SLERPI using machine learning, to define not only classification but also, diagnostic criteria, to aid in the early identification and prompt treatment of disease severity in SLE. SLERPI is a system that includes 14 clinical and serological features of SLE. These features have different weights and can be used to calculate personalized risk probabilities for SLE compared to other rheumatologic illnesses. The system provides categories of risk probabilities, such as "definite," "likely," "possible," and "unlikely," similar to clinical diagnostic reasoning. The simplified scoring system, with a threshold of > 7 out of a maximum score of 30.5, is derived from the original algorithm for diagnosing SLE. This simplified method has been designed to ensure accurate diagnosis of SLE, encompassing both early and severe cases, in daily practice. This study developed in Greek population showed that this tool had good diagnostic efficacy with a sensitivity of 94.2%, specificity of 94.4%, and precision of 94.2% [5]. In addition, they also confirmed the utility of SLERPI in different subphenotypes (i.e., lupus nephritis, neuropsychiatric lupus, hematological lupus, and severe SLE).

In the present study, the sensitivity 95.4% (95% CI 93.0–97.2) and the accuracy 94.1% (95% CI 92.3–95.6) was similar to the original SLERPI score, although the specificity 92.8% (95% CI 89.9–95.0) was slightly lower. In comparison to the three classification criteria, SLERPI exhibited the highest sensitivity, regardless of overt PolyA. ACR-1997 criteria demonstrated superior specificity at 98.8% (95% CI 97.3–99.6), whereas the SLICC-2012 criteria demonstrated the highest accuracy overall at 95.8% (95% CI: 94.3–97.1). In this line, a Chinese study showed that the ACR-1997 criteria exhibited the highest specificity at 96.4% (95% CI 94.0–98.0), whereas the SLICC-2012 criteria had the highest overall accuracy, reaching 95.0% (95% CI 93.1–96.4). SLERPI showed the highest sensitivity at 98.3% (95% CI 96.3–99.4) but the lowest specificity at 89.4% (95% CI 85.8–92.2) [7]. An Australian study corroborated these findings, reporting ACR-1997 criteria with the highest specificity at 95.9% (95% CI 90.8–98.7) and SLERPI with the lowest specificity at 84.6% (95% CI 76.9–90.4). The SLICC-2012 criteria also had the highest overall accuracy at 94.4% (95% CI 91.7–97.1), with both SLICC-2012 and SLERPI demonstrating excellent sensitivity at 98.5% (95% CI 96.7–99.4). Both studies found that SLERPI had the highest sensitivity but the lowest specificity [6]. Among the three studies, we had the lowest sensitivity.

This could be due to the presence of overt PolyA in the SLE group, although the sensitivity slightly decreased when these patients were removed. Other factors, such as ethnicity, could influence sensitivity. A recent study on the effects of ancestry, ethnicity, and gender, showed that there was a significantly greater proportion of Asian SLE patients with thrombocytopenia compared to non-Asian SLE [17]. SLERPI placed a greater weight on the presence of thrombocytopenia (4.5/7 score), and in our SLE group, only 10.3% of the patients presented this feature. Although there is another SLERPI study on the Colombian population, it exclusively compared the sensitivity among EULAR/ACR-2019, SLICC-2012, and SLERPI, which were 84.9%, 85.6%, and 89.0%, respectively [8]. The lower sensitivity observed in that study compared to the current one could be attributed to the sample size. Interestingly, ANA positivity, immunological disorders, and arthritis were the most prevalent features in both Colombian cohorts.

The present study identified 9 out of 430 patients in the control group with SLERPI scores greater than 7, who were misclassified with SLE. These patients had RA, SS, or AITD as their index disease and presented with features such as thrombocytopenia, proteinuria, leucopenia, malar rash, and ANA positivity. These characteristics, which overlap with those of SLE, may contribute to the decreased specificity of SLERPI. Zhang et al. [7] attributed the low specificity of SLERPI (89.4%, 95% CI 85.8–92.2) in the Chinese study to the predominance of SS and undefined connective tissue disease (UCTD) in the control group. In contrast, our control group included patients with RA, SSc, SS, AITD, and APS, and our specificity exceeded that reported in the Chinese study. The exclusion of individuals with SS and UCTD from the Chinese cohort significantly improved the performance of all classification criteria, particularly SLERPI. It has been noted that SS can precede SLE by 1–10 years [18]. Additionally, a cohort study of early UCTD reported that 8.3% of participants developed SLE after a 5-year follow-up [19].

Furthermore, Erden et al. [20] evaluated the specificity, rather than sensitivity, of SLERPI in 422 patients with UCTD. Among these, 39 patients were diagnosed with SLE, primarily due to the presence of thrombocytopenia, proteinuria, ANA positivity, and malar rash. SLERPI scores the presence of ILD as -1, counting against a diagnosis of SLE. While ILD was not observed in their SLERPI “SLE group,” it was present in six patients in the UCTD group, supporting the inclusion of ILD as a negative criterion [20]. All these findings support that SLERPI may serve as a predictive tool for identifying patients at risk of developing SLE.

In our study, SLERPI misclassified under the categories of unlikely (8 patients) and possible (9 patients) 17 SLE patients as false negatives. However, only seven of these patients did not meet any of the three other classification criteria. The majority of the misclassified patients (8/17, 47%) lacked a positive ANA, despite presenting with other symptoms consistent with SLE, such as classic malar rash, mouth ulcers, positive anti-dsDNA, and various hematological abnormalities. The remaining misclassified cases included patients with immunological disorders, inflammatory arthritis or cutaneous lupus who exhibited some, but insufficient, serological features to fulfill the criteria. Additionally, none of these patients exhibited thrombocytopenia or proteinuria.

The weighting importance of hematological disorders and proteinuria as laboratory items is demonstrated by Zhang et al. [21], who established that SLE can be recognized using laboratory items alone in patients with thrombocytopenia and nephritis. The ACR-1997 laboratory items criteria showed low sensitivity for both thrombocytopenia and lupus nephritis. In contrast, SLICC-2012, EULAR/ACR-2019, and SLERPI laboratory items criteria exhibited extremely high sensitivity, particularly the SLERPI laboratory items, which enabled the identification of all patients [21].

Additionally, our study only included adult patients, as clarified by the model's developers [22]. However, there is a study on a Turkish pediatric cohort showing that with the threshold set for adults, the SLERPI binary model yielded low sensitivity (90.0%) and specificity (81.2%). However, Batu et al. [23] suggest that setting the threshold at > 8 can make this model useful in pediatric practice. Our hypothesis is that such differences between age groups may be secondary to the duration of the disease. Furthermore, the small sample size could be the cause of these differences, given that other larger pediatric cohorts have evaluated ACR-1997, SLICC-2012, and EULAR/ACR-2019 with better performance [24].

Recognizing SLE promptly is essential for initiating treatment, particularly in hospitalized patients with severe disease. Early intervention in SLE is crucial for improving both short- and long-term outcomes. Kapsala et al. [25] showed that 87.4% of patients had an SLERPI score greater than 7 at the time of hospitalization, indicating a high probability of SLE. Patients not identified by SLERPI exhibited fever, thrombotic, or neuropsychiatric symptoms that the algorithm did not account for. By lowering the SLERPI threshold to 5 in patients with fever or thrombotic events, the diagnostic rate in this subgroup increased from 88.8% to 97.9%. However, including all neuropsychiatric events did not provide additional diagnostic value [25].

Existing criteria may fail to classify patients with SLE who have major organ disease, particularly neurological SLE. In our study, SLERPI showed excellent accuracy for identifying hematological lupus and lupus nephritis. Even though neuropsychiatric lupus has the lowest sensitivity at 93.2% (95% CI 85.7–97.5), it surpasses the 91.8% reported by Adamichou et al. (95% CI 82.2 – 96.5) [5].

Our results indicate that SLERPI has the potential to serve as a diagnostic and classification tool for SLE among Hispanics. It is worth mentioning that the comparison of the SLERPI, ACR-1997, SLICC-2012, and EULAR/ACR-2019 scales may have limits because the SLERPI is mostly focused on diagnosis, while the other two are more focused on classification. Additionally, the selection of classification criteria depends on the intended purpose. For research, it is crucial to have a uniform population that meets specified criteria, allowing for a better understanding of how different treatments affect patients and their prognosis. Studies can be observational or interventional, focusing on these specific patient groups. The primary purpose of classification criteria is to distinguish the target condition from other diseases and healthy individuals. Typically, when selecting classification criteria, high specificity is preferred over sensitivity to effectively differentiate clinically relevant illnesses. Numerous historical longitudinal cohort studies have employed the ACR-1997 criteria [6]. Given that the database for this study originates from a research center, our investigation has determined that the ACR-1997 criteria demonstrate the highest level of specificity compared to more recent classification criteria sets.

Study limitations must be acknowledged. This was a single-center study based on clinical records, that could have prone our study to reporting bias (e.g., on time from symptoms onset to consultation or clinical features). However, patients included in our database are systematically assessed for clinical serological manifestation of autoimmunity, including clinical features for other ADs (i.e., overt PolyA). Therefore, it is highly unlikely that such biases could have affected our results. In addition, given the retrospective nature of the study, we relied on the information registered in our database, which hindered the analysis of additional subgroups based on the severity and activity of the disease.

Although the study included a substantial number of participants (435 SLE patients and 430 controls), the distribution across different ADs in the control group was uneven, with some conditions having very few representatives (e.g., APS with only 1 patient). However, we conducted a randomized selection of participants from the main database built on the disease proportion of patients included. This reflects the real-world frequency of diseases in our population, and increases the external validity of this study. Other factors such as latent autoimmunity (i.e., positivity for autoantibodies not related to the index disease, and without fulfillment of classification criteria for any AD) were not evaluated and should be further considered in additional studies on this topic. Given that SLE affects 38.5% of African-Americans, 13.9% of Hispanics, 4.2% of Asians, 1.5% of Native Americans, and 36.2% of Caucasians [26], further research involving a broader range of ethnicities and larger sample sizes is necessary to validate the effectiveness of SLERPI in diagnosing SLE.

## Conclusion

SLERPI demonstrated high sensitivity and accuracy in diagnosing SLE, effectively distinguishing SLE patients from controls, regardless of the presence of overt PolyA. Compared to the ACR-1997, SLICC-2012, and EULAR/ACR-2019 criteria, SLERPI demonstrated greater sensitivity in the Colombian population, although it had lower specificity. The sensitivity of SLERPI remained robust across different SLE subphenotypes, particularly for hematological and nephritic forms, although it was less effective for neuropsychiatric lupus. Overall, SLERPI's performance was consistent regardless of overt PolyA status and comparable to established criteria, demonstrating its potential utility in the diagnosis and classification of SLE.

## Supplementary Information

Below is the link to the electronic supplementary material.

Supplementary file1 (DOCX 20 KB)

## Data Availability

Data will be available upon request to the corresponding author.

## Abbreviations

ACR: American College of Rheumatology
AD: Autoimmune disease
AIH: Autoimmune hepatitis
AIHA: Autoimmune hemolytic anemia
AITD: Autoimmune thyroid disease
ANA: Anti-nuclear antibody
APS: Anti-phospholipid syndrome
AUC: Area under the curve
CD: Crohn's disease
CI: Confidence interval
EULAR: European League Against Rheumatism
IQR: Interquartile range
ILD: Interstitial lung disease
MG: Myasthenia gravis
MS: Multiple sclerosis
PBC: Primary biliary cholangitis
PolyA: Polyautoimmunity
RA: Rheumatoid arthritis
ROC: Receiver operating characteristic
SCLE/DLE: Subacute cutaneous lupus erythematosus/discoid lupus erythematosus
SLE: Systemic lupus erythematosus
SLERPI: Systemic Lupus Erythematosus Risk Probability Index
SLICC: Systemic Lupus International Collaborating Clinics
SS: Sjögren's syndrome
SSc: Systemic sclerosis
T1DM: Type 1 diabetes mellitus
UCTD: Undefined connective tissue disease
