# Supplementary material for: Performance of the Systemic Lupus Erythematosus Risk Probability Index (SLERPI) in a cohort of Colombian population
Source: Clin Rheumatol. 2024 Sep 7;43(11):3313–22. doi: 10.1007/s10067-024-07108-x (PMC11489229; doi:10.1007/s10067-024-07108-x)
Supplement: Supplementary file 1 — Supplementary file1 (DOCX 20 KB) [file 10067_2024_7108_MOESM1_ESM.docx]

**Supplementary Table 1** Frequency of overt PolyA by index AD

| ADs | AITD  n: 43 (%) | APS  n:1 (%) | MS  n: 47 (%) | RA  n: 253 (%) | SLE  n:435 (%) | SS  n:56 (%) | SSc  n: 30 (%) |
| --- | --- | --- | --- | --- | --- | --- | --- |
| AIH | 0 (0.0) | 0 (0.0) | 0 (0.0) | 0 (0.0) | 2 (0.5) | 0 (0.0) | 0 (0.0) |
| AITD | 43 (100.0) | 0 (0.0) | 1 (2.1) | 21 (8.3) | 31 (7.1) | 9 (16.1) | 5 (16.7) |
| APS | 3 (7.0) | 1 (100.0) | 0 (0.0) | 0 (0.0) | 14 (3.2) | 0 (0.0) | 0 (0.0) |
| CD | 0 (0.0) | 0 (0.0) | 0 (0.0) | 0 (0.0) | 1 (0.2) | 0 (0.0) | 0 (0.0) |
| MG | 0 (0.0) | 0 (0.0) | 0 (0.0) | 0 (0.0) | 1 (0.2) | 0 (0.0) | 0 (0.0) |
| MS | 0 (0.0) | 0 (0.0) | 47 (100.0) | 0 (0.0) | 0 (0.0) | 0 (0.0) | 0 (0.0) |
| PBC | 0 (0.0) | 0 (0.0) | 0 (0.0) | 0 (0.0) | 1 (0.2) | 0 (0.0) | 0 (0.0) |
| RA | 7 (16.3) | 0 (0.0) | 0 (0.0) | 253 (100.0) | 10 (2.3) | 4 (7.1) | 4 (13.3) |
| SLE | 0 (0.0) | 0 (0.0) | 0 (0.0) | 0 (0.0) | 435 (100.0) | 0 (0.0) | 0 (0.0) |
| SS | 7 (16.3) | 0 (0.0) | 1 (2.1) | 12 (4.7) | 23 (5.3) | 56 (100.0) | 6 (20.0) |
| SSc | 4 (9.3) | 0 (0.0) | 0 (0.0) | 2 (0.8) | 3 (0.7) | 1 (1.8) | 30 (100.0) |
| T1DM | 1 (2.3) | 0 (0.0) | 0 (0.0) | 0 (0.0) | 2 (0.5) | 0 (0.0) | 0 (0.0) |

*AD* Autoimmune disease, *AIH* Autoimmune hepatitis, *AITD* Autoimmune thyroid disease, *APS* Anti-phospholipid syndrome, *CD* Crohn's disease, *MG* Myasthenia gravis, *MS* Multiple sclerosis, *PBC* Primary biliary cholangitis, *RA* Rheumatoid arthritis, *SLE* Systemic lupus erythematosus, *SS* Sjögren's syndrome, *SSc* Systemic sclerosis, *T1DM* Type 1 diabetes mellitus.
